# The Evolution of HIV Patient Retention and Care in French Guiana: A Broader View From the Système National des Données de Santé

**DOI:** 10.3389/fpubh.2022.823193

**Published:** 2022-02-17

**Authors:** Hélène Duplan, Sébastien Rabier, Christine Sudre, Leila Adriouch, Aude Lucarelli, Florence Huber, Louise Mutricy, Jean Marc Wojcik, Nicolas Vignier, Etienne Pascolini, Antoine Adenis, Mathieu Nacher

**Affiliations:** ^1^Direction du Service Médical de la Sécurité Sociale, Cayenne, French Guiana; ^2^COREVIH Guyane, Centre Hospitalier de Cayenne, Cayenne, French Guiana; ^3^General Medicine, Matoury, French Guiana; ^4^CIC INSERM 1424, Centre Hospitalier de Cayenne, Cayenne, French Guiana; ^5^Département Formation Recherche Santé, Université de Guyane, Cayenne, French Guiana

**Keywords:** HIV, cascade of care, follow-up interruption, private practice, French Guiana

## Abstract

**Background:**

Although the simplification of antiretroviral (AVR) treatment regimens and follow-up has led to fewer constraints for patients with HIV, their follow-up remains of paramount importance to optimize AVR therapy, to detect and prevent HIV-related morbidity, and prevent secondary infections. The problem of follow-up interruption in French Guiana has been persistent and seemingly impervious to efforts to alleviate it.

**Objective:**

The objective was to follow the trend of follow-up interruptions and to test the hypothesis that an increasing number of patients was, in fact, followed by private practitioners.

**Method:**

Using the complementary lenses of the hospital HIV cohort and the health insurance information system, we looked at the incidence of follow-up interruption and the proportion of patients followed by private practitioners.

**Results:**

We tallied 803 persons that were not known to have died and who were lost to follow-up. Over time, hospital outpatients were lost to follow-up significantly sooner. By contrast, there was a significant trend with more and more patients exclusively followed by private practitioners.

**Conclusion:**

While hospital outpatient care remains by far the most common mode of patient care, there seems to be a gradual erosion of this model in favor of private practice.

## Introduction

French Guiana is the French overseas territory where the HIV epidemic is most prevalent (1). Recent modeling using the European Center for Disease Control (ECDC) modeling tool estimated the number of persons living with HIV to be over 3,200, 3,000 of whom knew their diagnosis (2). HIV transmission in French Guiana is mostly heterosexual and three of four patients are foreign citizens (3). French Guiana has the highest gross domestic product (GDP) per capita in South America and, as such, it is attractive for the poor Caribbean and South American populations in search of a better life. In a context of precariousness and sexual vulnerability, a large proportion of infected immigrants actually acquire the virus after their arrival in French Guiana (4, 5). The standards of health care are those of mainland France. Since 2013, antiretroviral (AVR) treatment is recommended for all patients irrespective of their CD4 count. All persons living with HIV receive free or paid AVR treatments regardless of their origin or socio-economic level. Undocumented immigrants with HIV are eligible for residence permits for medical reasons and health insurance coverage. Although the simplification of treatment regimens and follow-up has led to fewer constraints for the patients, their follow-up remains of paramount importance to optimize anti-retroviral therapy to detect and prevent HIV-related morbidity and prevent secondary infections. In 2006, we had studied follow-up interruption and its risk factors in French Guiana (6). Younger patients, foreigners, untreated patients, and non-immunosuppressed patients at the time of diagnosis were more likely to interrupt follow-up. In addition, the greatest risk of interruption was usually within the first 6 months after diagnosis. Since then, nearly all patients are given AVRs with regimens that are more potent but better tolerated, which usually simplifies medical follow-up. In the post 90-90-90 context, French Guiana aims to optimize the cascade of care by improving early testing and treatment and retaining patients in the healthcare system so they can continue to benefit from virological suppression. Despite progress, this remains a problem in France (7, 8). For the specific case of French Guiana, our hypothesis was that different and novel forces were at play and may have modified the adherence to outpatient follow-up. First, in the widespread early treatment with powerful and well-tolerated drugs, the simplification of follow-up would predict that patient retention should increase over time. Second, because patient follow-up is much simpler than before, general practitioners are more inclined to accept such a task, and patients –always weary of being spotted in a specialized HIV outpatient care center—may prefer to be followed closely to their home, in the “stigma-free” practice of their family physician. We therefore looked at the proportion of patients lost to follow-up –from the point of view of hospital outpatient care in different time periods. We also looked, for the first time, at the data from the health insurance system (9), which notably reimburses all antiretroviral drugs (ARVs) to determine the proportion of patients for whom ambulatory care was given from a specialized hospital department and those who only received ambulatory care from a private practitioner.

## Methods

### Patients From the Hospital Outpatient Clinics

The information systems for HIV in French Guiana, mandatory reporting of new HIV infections and AIDS cases, and the French Hospital Database of HIV, a cohort for which specifically trained research technicians have collected data on HIV patients since 1989 {first in the DMI2 government program until 2008, then in eNADIS/DATAIDS [Dat'AIDS cohort (clinicaltrials.gov ref. NCT02898987)]}, were used for the study.

### Lost to Follow-Up

This cohort allowed us to obtain the number and proportion of patients lost to follow-up (not having consulted in more than 12 months) from the point of view of the hospital system.

### Determination of the Proportion of Patients Receiving Treatment in a Public Hospital or in a Private Practice

Since 2017, health administrations and researchers may, with permission, access data from the Système National des Données de Santé which now combines health care reimbursement data (SNIIRAM), hospital data (PMSI), and death certificate data (CepiDC). For French Guiana, this allowed us to obtain, for the first time, the proportion of persons treated for HIV in a public hospital structure or in private practice, or both.

### Selection of ARV Treatments

The ARV treatments were selected from the code identifiant de présentation (CIP) code of the Drug Database [base de données publique des médicaments (BDM)], the reference database of drugs reimbursed by the French Health Insurance.

### Patient Selection

The data used to select the people on ARVs were taken from the inter-scheme consumption datamart (DCIR). All the health insurance beneficiaries were included in the study if they received at least one reimbursement for the delivery of ARVs during the year, even when the treatments were prescribed and/or delivered outside French Guiana. The specific insurance regimens included the General Health Insurance Scheme, including beneficiaries of the free complementary health insurance coverage for the poor French residents or legal migrants (CMUC/CSS) and the state medical aid for undocumented foreigners (AME); the social security system for the self-employed (RSI); and the agricultural social mutual insurance (MSA) of French Guiana.

A subsample was also made up of patients who received at least one reimbursement for ARVs under the AME or CMUC/CSS during the year. Since AME or CMUC/CSS reflect poverty, these selection criteria allowed to test the hypothesis that trends for private or public follow-up differed between socially precarious and non-precarious populations.

### Description of the Evolution of the Type of Care

The type of care was defined as follows: exclusively hospital-based when the ARV treatments for which the patient was reimbursed during the year were exclusively prescribed by the hospital sector, exclusively private when the ARV treatments for which the patient was reimbursed during the year were exclusively prescribed by the private sector and mixed when the ARV treatments for which the patient was reimbursed during the year are prescribed by the hospital sector and the private sector.

The annual evolution of the distribution of the type of care was studied over the 2016–2020 period in the population of all patients on ARVs at the regional level and in the population, including only CMUC/CSS or AME patients (precarious persons) at the regional level.

### Data Analysis

Using the DAT'AIDS cohort data, single failure survival analyses were performed with follow-up interruption of more than 12 months (not due to death) as a failure event for a patient without knowledge of his or her present follow-up status. Kaplan Meier curves were plotted. Log Rank tests were performed. These analyses allowed us to quantify and visualize the time until follow-up interruption for different periods. For the health insurance data, a linear trend Chi-square test was used to test the statistical significance of the annual change in the different types of care, observed graphically over the period studied. This trend analysis aimed to determine whether the process was gradual.

### Regulatory and Ethical Issues

Anonymized individual data from the DAT'AIDS database was used (clinicaltrials.gov ref. NCT02898987). For the proportion followed up in private or public structure, the anonymized data from the système national des données de santé (SNDS) is accessible for certain organizations with a public service mission. Particularly, these organizations, listed by decree by the Conseil d'Etat, issued after the opinion of the Commission Nationale Informatique et Libertés (CNIL), may access certain data on a permanent basis in order to carry out their missions. This is the case for researchers of Academic hospitals and the Institut National de la Santé et de la Recherche Médicale (Inserm).

## Results

### Hospital Follow-Up in Time

**Figure 1:**
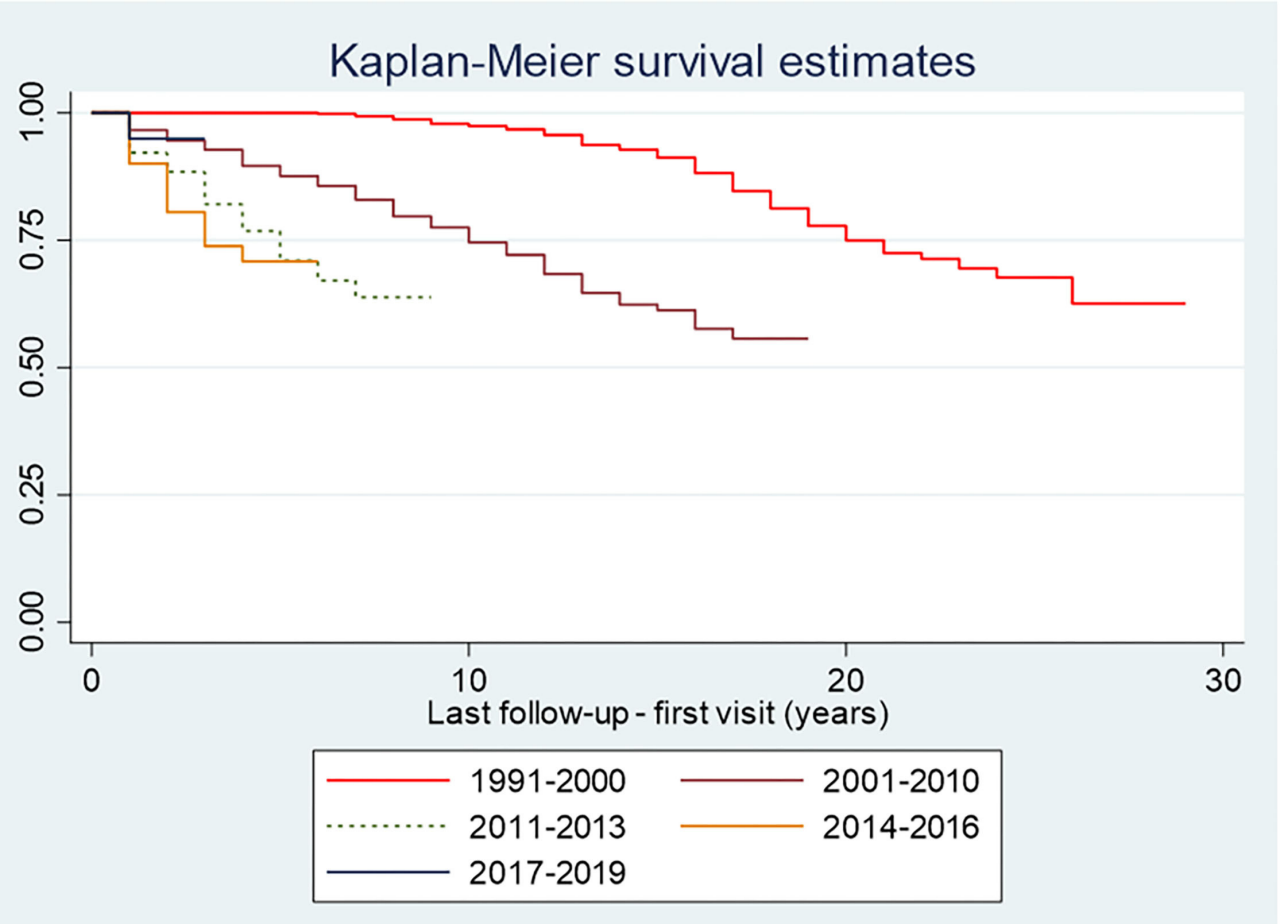
Incidence of follow-up interruption in French Guiana from the point of view of the hospital HIV outpatient cohort.

We tallied 803 persons that were not known to have died and who were lost to follow-up. Figure 1 shows that over time, patients seemed to be lost to follow-up increasingly sooner (Log-rank test, *p* < 0.001, Table 1).

**Table 1:** Log-rank test comparing the incidence of follow-up interruption for different consecutive years.

**Period**	**Events observed**	**Events expected**
2016	129	281.82
2017	417	402.84
2018	151	83.48
2019	157	74.52
2020	22	33.33
*Total*	***876***	***876.00***

*CHI2 (4) = 307.45; p = 0.0000*.

### The Total Number of Persons on ARV in French Guiana

When looking at the health insurance data, in 2020, there were 3,047 patients who benefitted from health insurance payments for ARV treatment. This total number has been steadily increasing since 2016 (see Table 2). The evolution rate over the period was 33.35% with an average annual evolution rate of 7.46%. However, the evolution of the number of patients was more moderate in 2020, a year when activity was completely disorganized by coronavirus disease 2019 (COVID-19) and lockdowns.

**Table 2:** Distribution of the type of antiretroviral (ARV) follow-up (private, hospital, or mixed) in Guiana between 2016 and 2020.

**Year**	**Private practice follow-up *N* (%)**	**Hospital follow-up *N* (%)**	**Mixed follow-up *N* (%)**	**Total number of patients followed**	**Annual rate of change (%)**
2016	245 (10.72)	1,449 (63.41)	591 (25.86)	2,285	
2017	251 (10.33)	1,604 (66.01)	575 (23.66)	2,430	6.35
2018	320 (12.01)	1,607 (60.32)	737 (27.67)	2,664	9.63
2019	359 (12.26)	1,758 (60.04)	811 (27.7)	2,928	9.91
2020	497 (16.31)	1,750 (57.43)	800 (26.26)	3,047	4.06

*Source: SNDS/DCIR—Beneficiaries of the RG (CMU/CSS and AME)*.

*RSI and MSA affiliated in French Guiana, excluding complementary insurances not managed by the computer*.

### Hospital and/or Private Follow-Up

In 2020, 57.43% (*n* = 1,750) of patients were followed *exclusively* by the hospital sector. The proportion of patients treated *exclusively* in the private sector was 16.31% (*n* = 497), and that of patients with mixed treatment prescription (private and hospital) was 26.26% (*n* = 800) (Table 1).

Until 2019, the number of patients followed up increased whatever the type of care. In 2020, only the number of patients followed up exclusively in the private sector continued to increase (+138 patients), whereas the number of patients followed up in the hospital sector or with mixed follow-up decreased slightly (see Table 2). Furthermore, the proportion of patients followed up *exclusively* by the hospital sector has decreased over the last 3 years, particularly in 2020. The change over the entire period was statistically significant (*p* < 0.0001). Conversely, the proportion of patients followed exclusively by the private sector has been steadily increasing since 2018 (the trend was statistically significant; *p* < 0.0001). Finally, the proportion of patients with follow-up in both the hospital and private sectors has varied little since 2016. However, there was a slight decrease in 2020 compared to 2018 and 2019, years in which the proportion had stabilized at around 28% of patients (the trend over the whole period being statistically significant; *p* = 0.006). Because COVID-19 in 2020 led to dramatic changes (lock-downs, cancelation of hospital consultations, etc.), we also computed the trends for 2016–2019, and the observation of a linear increase of the trend toward outpatients was still significant: *p* = 0.004 and *p* = 0.002, respectively.

*Focus on patients with indicators of precariousness (*Couverture Maladie Universelle Complémentaire/Complémentaire Santé Solidaire or Aide Médicale Etat).

In 2020, 1,873 patients who had been reimbursed for ARVs were beneficiaries of the CMUC/CSS or the AME (i.e., 61.47% of all patients on ARVs) insurance plans for socially precarious persons. The number of patients in this situation increased until 2019 but decreased in 2020. Overall, the increase over the whole period was 24.53%, with an average annual growth rate of 5.64%. In 2020, 1,082 of the 1,873 patients receiving CMUC/CSS or AME on ARVs had *exclusive* hospital follow-up (i.e., 57.77%). This proportion steadily decreased over the last 3 years after an initial increase in 2017 (the change over the whole period being statistically significant; *p* < 0.0001). Conversely, the proportions of patients managed *exclusively* by the private sector or benefiting from mixed public and private follow-up, after having decreased over the year 2017, increased over the years 2018, 2019, and 2020. The proportion of precarious patients on ARVs exclusively followed up in the private sector thus rose to reach 14.15% in 2020 (a statistically significant trend for the period 2016–2020; *p* < 0.0001). The proportion of patients with mixed management (combination of public and private follow-up) increased by + 2 points between 2016 and 2020 (the trend was statistically significant over the period; *p* = 0.0036) to reach more than 28% in 2020 (Table 3).

**Table 3:** Distribution of the type of antiretroviral follow-up (private practice, hospital, or mixed) in French Guiana between 2016 and 2020 among precarious patients.

**Year**	Private follow-up *n* (%)^*^	**Hospital follow-up *n* (%)**	Mixed follow-up *n* (%)^**^	**Total number of patients followed**	**Annual rate of change (%)**
2016	137 (9.11)	974 (64.76)	393 (26.13)	1,504	
2017	140 (8.98)	1,064 (68.25)	355 (22.77)	1,559	3.66
2018	185 (10.61)	1,078 (61.85)	480 (27.54)	1,743	11.8
2019	210 (10.97)	1,178 (61.51)	527 (27.52)	1,915	9.87
2020	265 (14.15)	1,082 (57.77)	526 (28.08)	1,873	−2.19

*Source: SNDS/DCIR—Beneficiaries of Couverture Maladie Universelle Complémentaire/Complémentaire Santé Solidaire and Aide Médicale Etat in French Guiana*.

* *Linear trend chi2 (private vs. hospital only, 2016–2020): p < 0.001*;

** *Linear trend chi2 (mixed private+hospital vs. hospital only, 2016–2020): p = 0.003*.

*Because of trend distortions resulting from COVID-19, the trend was recalculated for 2016–2019, excluding 2020*.

*Linear trend chi2 (private vs. hospital only, 2016–2009): p = 0.004; **Linear trend chi2 (mixed private+hospital vs. hospital only, 2016–2020): p = 0.002.

## Discussion

For the first time, the use of health insurance data allowed us to see the whole picture of HIV care in French Guiana. The actual number of persons benefiting from antiretroviral treatment in French Guiana was actually very close to the recent estimations using HIV-cohort data for the ECDC modeling tool (1). The number of persons on AVR treatment has substantially increased over the study time but slowed down in 2020. Given the simultaneous disruption of information systems and HIV testing and care, it is difficult to tease out whether this reflects the slowing down of an epidemic where increasing numbers of persons are virologically controlled, or the COVID-19-related gap in new diagnoses and patient referrals. Data from 2021 is also likely to be impacted by COVID-19, so only future post-COVID-19 years may allow us to find out a better explanation for our observation.

While the organization of care and virological results had gradually improved over the years, we were initially surprised to see the rate of persons lost to follow-up from the hospital outpatient cohort actually increased over time. However, clinicians had perceived the feedback from patients increasingly tempted by a follow-up by their private practitioner for proximity reasons and because going to specialized HIV outpatient departments was more stigmatizing than a waiting room of a general practitioner. At the same time, treatment regimens and follow-up are far simpler than they used to be, which expands the number of physicians willing to take-on the task of being the referent physician of patients with HIV. Furthermore, the aging HIV-cohort (39.5% aged over 50 years) increasingly requires general practitioners–not infectious disease specialists—to manage comorbidities, which may be another incentive to have an integral follow-up by one's family physician instead of a specialist. This perception seems to be vindicated by the health insurance data, which shows a growing proportion of persons choosing to be exclusively treated by their private practitioner. Furthermore, about a quarter used both the private and public sectors, suggesting pragmatic convenience was behind this behavior. The COVID-19 disruption of HIV follow-up may have further tilted follow-up toward private practice in 2020, and it remains to be seen whether this will be corrected in the future or if it has accelerated an ongoing trend toward decentralization of HIV care.

At first view, one could have hypothesized that this trend toward private practice did not include the most precarious cases which in French Guiana's HIV cohort, represents 2/3rds of patients if we only look at the type of Health Insurance. However, this was refuted by the data which shows the exact same trend among the more precarious and shows that, contrary to widespread beliefs, poor populations do have access to private practice and use it readily as soon as they have health coverage. Since 2000, the Public Health Law has ensured that the most precarious cases do not have to advance any fees for medical consultations, and thus there are no financial obstacles to consult a private practitioner instead of a hospital physician.

The study has a number of limitations. First, the use of health insurance regimens as a proxy for poverty is legitimate, but it may actually underestimate the degree of precariousness. Particularly, the SNDS did not provide other indicators, such as country of birth or nationality, which may have served as another proxy for precariousness. Another limitation is that the simultaneous accelerating rate of persons being lost to follow-up from the hospital system and the increasing proportion of patients exclusively followed by private practitioners do not formally prove that those that do not come back in the outpatient ward are actually consulting private practitioners. Instead, they could have left the territory, or stopped treatment and follow-up, or died. However, for deaths or treatment interruptions, this seems largely unlikely because opportunistic infections and deaths –which eventually are counted—have substantially decreased over time suggesting that indeed many of the patients who do not come back to the hospital outpatient clinic are receiving care in private practices.

In conclusion, while hospital outpatient care remains by far the most common mode of patient care, there seems to be a gradual erosion of this model in favor of private practice. For the first time, the Health Insurance information system allows obtaining a broader view of patient care than hospital cohort data alone. In the rapidly evolving therapeutic and conceptual landscape of HIV care, in the context of aging cohorts and shifting patient preferences, coordinating continuous medical education with private practitioners seems to be crucially important so that every caregiver is up to date with the current guidelines. This slow gradual process may even have accelerated in the transformational context of the COVID-19 pandemic.

## Data Availability Statement

The data analyzed in this study is subject to the following licenses/restrictions: The DATAIDS data may be shared upon reasonable request. The health Insurance data requires additional permissions. Requests to access these datasets should be directed to corevih@ch-cayenne.

## Ethics Statement

The studies involving human participants were reviewed and approved by Commission Nationale Informatique et Libertés. The patients/participants provided their written informed consent to participate in this study.

## Author Contributions

MN: study design. HD and NV: data analysis. HD and MN: first draft writing. CS, LA, AL, FH, LM, JW, NV, EP, and AA: review and editing. All authors contributed to the article and approved the submitted version.

## Conflict of Interest

The authors declare that the research was conducted in the absence of any commercial or financial relationships that could be construed as a potential conflict of interest.

## Publisher's Note

All claims expressed in this article are solely those of the authors and do not necessarily represent those of their affiliated organizations, or those of the publisher, the editors and the reviewers. Any product that may be evaluated in this article, or claim that may be made by its manufacturer, is not guaranteed or endorsed by the publisher.
